# Evaluation of microbiological, chemical, and sensory properties of cooked probiotic sausages containing different concentrations of astaxanthin, thymol, and nitrite

**DOI:** 10.1002/fsn3.2000

**Published:** 2020-11-12

**Authors:** Issa Mohammadpourfard, Ali Khanjari, Afshin Akhonzadeh Basti, Carlos Herrero‐Latorre, Nabi Shariatifar, Hedayat Hosseini

**Affiliations:** ^1^ Department of Food Hygiene and Quality Control Faculty of Veterinary Medicine University of Tehran Tehran Iran; ^2^ IAQBUS‐Institute of Research on Chemical and Biological Analysis Dpto. Química Analítica Nutrición y Bromatología Facultad de Ciencias Universidade de Santiago de Compostela Lugo Spain; ^3^ Department of Food Safety and Hygiene School of Public Health Tehran University of Medical Sciences Tehran Iran; ^4^ Department of Food Science and Technology National Nutrition and Food Technology Research Institute Faculty of Nutrition Sciences and Food Technology Shahid Beheshti University of Medical Sciences Tehran Iran

**Keywords:** astaxanthin, *Bacillus coagulans*, *Clostridium perfringenes*, cooked beef sausage, thymol

## Abstract

In this study, the effects of different concentrations of thymol and astaxanthin on control of *Clostridium perfringenes* and also microbial, chemical, and organoleptic properties of common and probiotic beef cooked sausages containing two levels of nitrite during storage at refrigerated condition during 45 days were evaluated. Based on findings, control group had significantly higher total volatile base nitrogen (TVB‐N) than nitrite‐, thymol‐, and astaxanthin‐treated samples. At the end of the storage time in control, thiobarbituric acid reactive substances (TBARS) value reached 1.96 mg/kg, while the values for treated samples remained lower than 1.63 mg/kg. Final count of lactic acid bacteria decreased approximately 1.67–3.79 log CFU/g in treated samples compared with the control group (*p* < .05). A reduction between 1.46 and 2.46 log CFU/g in *C. perfringenes* count was recorded for the treated samples in comparison with control group after 45 days of storage.

## Graphical abstract

In heat‐treated meat products such as cooked sausage, probiotics are generally not used because of adverse effect of thermal treatment on their viability and stability. One of the novel techniques to improve nutritional attributes of meat products is integration of Bacillus coagulans, a thermophilic probiotic microorganism. Using some natural derived additives and preservatives such as astaxanthin and thymol is a novel approach to decrease the use of nitrite and extend the shelf‐life of cooked sausages and their safety.

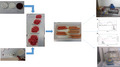

## 1 INTRODUCTION

Cooked sausages belong to popular ready‐to‐eat products usually produced from different kinds of fresh meats (Khodayari et al., 2019; Mendoza et al., 2001). However, due to the potential health risk, concerns about chemical food preservatives and additives have been increased (Campêlo et al., 2019; Lee et al., 2019; Lee et al., 2017). In general, consumers prefer minimally processed meat products with natural food additives (such as annatto, saffron, paprika, and astaxanthin) and natural food preservatives (such as pediocin, natamycin, lactoferrin, and thymol) (Campêlo et al., 2019; Carballo, Mateo, et al., 2019; Karam et al., 2019). Therefore, the production of safe and high‐quality meat products, using natural green additives and preservatives, has increasingly gained interest in food science research area.

*Clostridium perfringenes* is one of the most important foodborne pathogens that leads to severe infection and even death. It was documented that vegetative cells of *C. perfringens* are susceptible to heat. Unfortunately, it is difficult to inactivate spores of *C. perfringens*, because of their resistance to heat (survive at 100°C for ≤1 hr). Therefore, it is necessary to find new methods to control *C. perfringens* in meat products (Juneja, Baker, Thippareddi, Snyder Jr, & Mohr, 2013; Lee et al., 2019; Limbo et al., 2010).

Astaxanthin, as a keto‐carotenoid, seems to be a very promising novel natural additive with a high potential application in food processing. Recent data reveal that the addition of astaxanthin to food, directly and indirectly, improves its oxidative stability (Carballo, Giráldez, et al., 2019). Furthermore, it has been declared that astaxanthin has beneficial effects in the prevention and treatment of many diseases, such as chronic inflammatory diseases, metabolic syndromes, and certain cardiovascular, gastrointestinal, and liver diseases (Leite et al., 2010; Naito et al., 2004; Uchiyama et al., 2002). Astaxanthin is categorized as GRAS (Generally Recognized As Safe) by the US Food and Drug Administration (USFDA), and it has been considered as safe additive by the European Union Food Safety Authority (EFSA). Applying this compound as an additive or a packaging component can be introduced as a novel constitutes in meat products (Yang et al., 2013).

Thymol, also recognized as 2‐isopropyl‐5‐methylphenol, a dietary monoterpene phenol, is one of the dominant components in thyme species. For centuries, plants containing thymol in their constituents have been used in traditional medicine (Meeran et al., 2017). Thymol exhibits potent antimicrobial and antioxidant activities that is frequently used in food industry as a natural preservative (Karam et al., 2019; Meeran et al., 2017).

Nitrite is an additive commonly utilized in the preparation of sausages; this compound prevent the lipid oxidation, impeding the development of rancid off‐flavors, producing pink color, and inhibiting the growth of spoilage and pathogenic bacteria, especially *Clostridium* spp. (Choi et al., 2017; Lee et al., 2019; Xiang et al., 2019). However, it has been demonstrated that high intake of nitrite results in a risk to human health as this compound is a well‐known precursor of N‐nitroso compounds classified as potent human carcinogens (Ma et al., 2018; Šojić et al., 2019). For this reason, the reduction or elimination of the use of nitrite in meat products is highly desirable. Therefore, research and evaluation of natural alternatives have been taken for the reduction of nitrite addition in meat products.

Probiotics are live microorganisms that is its sufficient administration has beneficial health effects such as increasing nutritional value of food, and improving immune system and digestive system function of the host. Although probiotics are mostly administered through dairy products, meat products can also be used as a probiotic carrier (Chugh & Kamal‐Eldin, 2020; Khaledabad et al., 2020; Ryan et al., 2020; Zendeboodi et al., 2020; Zhu et al., 2020). In heat‐treated food products, probiotics are generally not used because of negative effect of thermal treatment on their viability and stability. Integration of thermophilic probiotic microorganisms into meat products is one of the novel techniques. Hence, *Bacillus coagulans* can be used as probiotic due to their heat‐resistant spore forms (Konuray & Erginkaya, 2018).

*Clostridium perfringenes* is one of the most important foodborne pathogens that lead to severe infection and even death. It was documented that vegetative cells of *C. perfringens* are susceptible to heat. Unfortunately, it is difficult to inactivate spores of *C. perfringens*, because of their resistance to heat (survive at 100°C for ≤1 hr). Therefore, it is necessary to find new methods to control *C. perfringens* in meat products (Juneja et al., 2013; Lee et al., 2019; Limbo et al., 2010).

Taking into account the above explanations, the aim of the present work was to study the effect of the use of astaxanthin and thymol in relation to the reduction of nitrite in cooked probiotic sausages and to evaluate their influence on chemical, microbial, and sensory quality of the product and also the growth of *C. perfringenes* during the storage time.

## 2 MATERIALS AND METHODS

### 2.1 Sausage samples preparation

Cooked sausages in the present study were produced according to the traditional preparation of Iranian meat product factories (three replicates). Their ingredients included beef, 65%; ice, 13.5%; oil (Aceites de Las Heras Co., Valencia, Spain), 9%; starch (Panreac Quimica S.L.U., Barcelona, Spain), 2.5%; soy protein isolate (Enerzona Co, Italy), 2.5%; whole egg, 3%; dried milk (Panreac Quimica S.L.U., Barcelona, Spain), 2.5%; sucrose (Guinama Co., Valencia, Spain), 0.75%; sodium chloride (Merck, Germany), 0.3%; garlic powder, 0.2%; nutmeg 0.1% and black pepper, 0.1%; ascorbic acid (Panreac Quimica S.L.U., Barcelona, Spain), 0.3%; and sodium polyphosphate (Panreac Quimica S.L.U., Barcelona, Spain), 0.25%. Beef was obtained from a beef processing center (Novafrigsa, Lugo, Spain), and spices were bought from La Hierbas a granel (Lugo, Spain). Based on this recipe, 13 various formulations were prepared with or without *B. coagulans* GBI‐30 6086 (obtained from Department of Food hygiene, Faculty of Veterinary Medicine, University of Tehran, Tehran, Iran) and different amounts of astaxanthin (AstaReal®EL25, Nacka, Sweden), thymol (Sigma‐Aldrich, USA), and nitrite (Sigma‐Aldrich, USA), as indicated in Table 1. Formula 1 was used for the control group, and the remaining twelve formulations were prepared with addition of different quantities of nitrite (60 and 120 ppm), astaxanthin (0, 300, and 450 ppm), and thymol (125 and 250 ppm). For preparing the inoculated formulations, spores of *B. coagulans* (12 log CFU/kg) were inoculated at the start of cutter step after adding 50% of water and then mixing the sausage emulsion for 3 min. Then, half of emulsion of sausage samples were inoculated with culture of *C. perfringens* type A ATCC 13124 (obtained from Pasteur Institute of Iran) and mixed with another 2 min at 230 rpm (the final concentration of *C. perfringens* was approximately 6 log CFU/g). After merging/chopping step, all batches were stuffed into edible collagen envelope (diameter of 21 mm). Then, they were vacuumed and cooked (at 80°C for 60 min), and after heat process, sausages were cooled in water bath for 45 min and stored at 4°C until analysis.

**Table 1:** Composition of the different sausage formulations

Formula	*Bacillus coagulans* GBI−30 6086 addition	Nitrite (mg/kg)	Thymol (mg/kg)	Astaxanthin (mg/kg)
1 (control)	Without	0	0	0
	With	0	0	0
2	Without	60	125	0
	With	60	125	0
3	Without	60	125	300
	With	60	125	300
4	Without	60	125	450
	With	60	125	450
5	Without	60	250	0
	With	60	250	0
6	Without	60	250	300
	With	60	250	300
7	Without	60	250	450
	With	60	250	450
8	Without	120	125	0
	With	120	125	0
9	Without	120	125	300
	With	120	125	300
10	Without	120	125	450
	With	120	125	450
11	Without	120	250	0
	With	120	250	0
12	Without	120	250	300
	With	120	250	300
13	Without	120	250	450
	With	120	250	450

### 2.2 Microbiological and chemical procedures

#### 2.2.1 Microbiological analysis

Samples (25 g) were aseptically transferred to a stomacher bag and homogenized with 225 ml sterile peptone water (0.1% w/v) for 3 min. From each homogenate sample, appropriate serial dilutions were prepared in peptone water (0.1% w/v). Lactic acid bacteria (LAB) were enumerated on De Man Rogosa Sharpe Agar (MRS Agar) following incubation in anaerobic conditions at 30 ºC for 72 hr. Yeasts and molds were counted on Dichloran Rose‐Bengal Chloramphenicol Agar (DRBC Agar), after incubation at 25°C for 5 days. The count of vegetative cells and spores of *C. perfringens* were analyzed during the storage period. *C. perfringens* were enumerated on tryptose–sulfite–cycloserine agar (TSC agar) following incubation under anaerobic conditions according to the method used by Lee et al. (2019) (Lee et al., 2019). Total *Enterobacteriaceae* count were determined using Violet Red Bile Glucose Agar (VRBG Agar) which were incubated at 37°C for 24–48 hr. Data were recorded and then converted as log (CFU/g). In order to enumerate *B. coagulans* spore count, appropriate serial dilutions were exposed to heat shock at 80°C for 15 min. Afterward, cultivated on Tryptic Soy Agar (TSA) which was incubated at 37°C for 48 hr according to the method recommended by Somavat et al. (2013) (Somavat et al., 2013). *Staphylococcu saureus* detection was done, using International Organization for Standardization (ISO) 6888–3:2003 method.

#### 2.2.2 Chemical analysis

Moisture, protein, and fat were determined at the last day of storage according to the Official Methods of Analysis of AOAC International (Latimer & George Jr, 2016). TBARS (2‐thiobarbituric acid reactive substances) and TVN (total volatile nitrogen) content of sausage samples were determined according to the method described by Alirezalu et al. (Alirezalu et al., 2019). For the pH measurements, the sample was homogenized and diluted with distilled ultrapure water at 1:10 ratio, and then, pH was measured using a HI221 Calibration Check Microprocessor pH meter (Hanna Instruments S.L., Spain).

### 2.3 Sensorial evaluation

For assessing sensorial characteristics, sausage samples were evaluated at the end of the storage by an organoleptic panel conformed of trained members. Briefly, the panelists (5 people) were asked to score color, odor, flavor, and overall acceptance and desirability of sausage samples. Samples were evaluated according to the nine‐point hedonic scale, from 0 (dislike extremely) to 9 (like extremely), and considering 5 as stands for limit of acceptance. Samples were labeled with 3‐digit random numbers to avoid identification, and they were presented to the panelist in random order.

### 2.4 Statistical and chemometrics procedures

Data were analyzed through one‐way analysis of variance (ANOVA) test; multiple comparisons were carried out by Tukey's tests using IBM SPSS Statistics v.19 software (International Business Machines Corporation, Armonk, NY, USA). In all cases, *p* < .05 was considered statically significant.

In order to study the latent relationships (between samples and between variables) residing in the data for the different sausage formulations two display chemometric techniques that were applied to the obtained microbiological and chemical data include principal components analysis (PCA) and hierarchical cluster analysis (HCA). PCA is a chemometric procedure frequently applied for obtaining a primary multidimensional evaluation of the data set and for reducing the data dimension with minimal loss of useful information. PCA was done by decomposing the original data matrix as a product of two other matrices. The first one (*score* matrix) contains information about the samples, while the second matrix (*loading* matrix) includes information related to the variables. When the number of principal components considered to analyze the problem is smaller than the number of original variables, PCA simplifies the dimension of the problem and this allows the appropriate study of the original data matrix *X* in a reduced space (Jolliffe, 1986). HCA was used as a second display chemometric technique. This technique (often used jointly or complementarily with PCA to study the internal structure of a data set) is generally applied to the data matrix to search for natural groups of samples (or variables) on the basis of their distance in the multidimensional space. In the present study, sausage samples of the different formulations were hierarchically clustered according to the squared Euclidean distances between them. Clusters were calculated on the basis of agglomerative Ward's method (Massart, 1983). This chemometric technique produced a graphical output called dendrogram: a tree diagram frequently used to illustrate the arrangement of the clusters produced by HCA. Both chemometric techniques PCA and HCA were performed using Statgraphics Centurion XVI V.16.1.15 (Statistical Graphics Corporation, Rockville, MD, USA).

## 3 RESULTS AND DISCUSSION

Beef cooked sausages as proteinaceous products are one of the popular meat products all over the world. Natural green preservatives (thymol and astaxanthin) could be used to enhance the shelf life and safety of these products.

Cooked probiotic meat products can be used to transmit probiotic bacteria. In addition, some researchers believe that the sausage matrix protects the survival of probiotic through the gastrointestinal tract. *B. coagulans* partially, and sodium nitrite substitutes in probiotic sausages, can produce approximately the same amount of myoglobin nitrosyl which may be present due to nitrite reductase activity in the microbial strain, which can reduce nitrite(De Vuyst et al., 2008; Zhu et al., 2020).

### 3.1 Microbiological analysis

**Figure 1:**
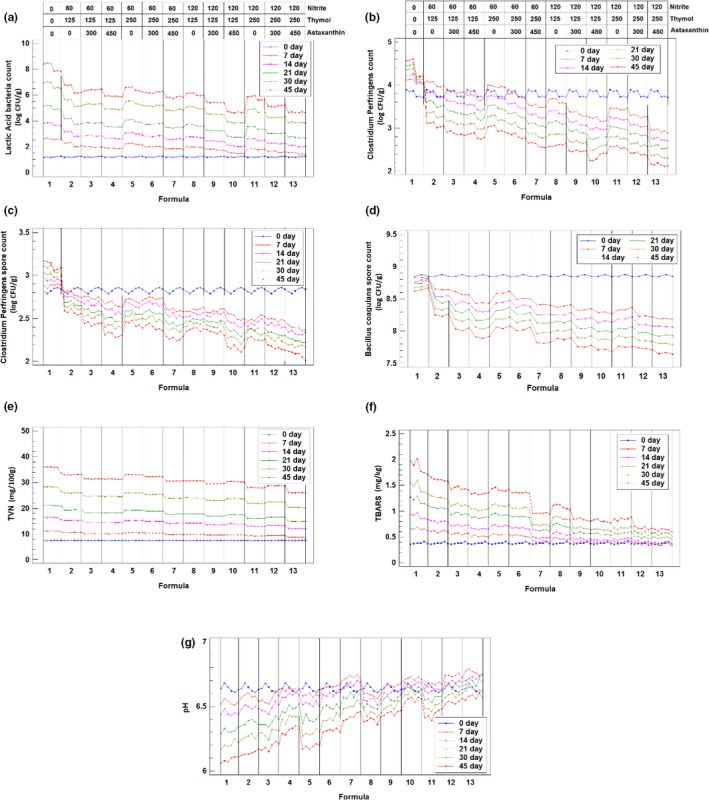
Evolution of different parameters analyzed along storage period: [a] lactic acid bacteria (log CFU/g); [b]*Clostridium perfringenes*(log CFU/g); [c]*C. perfringenes*spores (log CFU/g); [d]*Bacillus coagulans*spore count (log CFU/g); [e] TVN (mg/100 g); [f] TBARS (mg/kg); and [g] pH

Microbiological analysis was done during the storage period (at 0, 7, 14, 21, 30, and 45 days) for different formulations of sausage samples prepared; as indicated in section 2.1, they contain different concentrations of thymol, astaxanthin, and nitrite. The results presented in Figure 1 include those obtained for the six replicate samples studied for each formulation (three without *B. coagulans* and three with *B. coagulans* addition). The bacteriological changes in the cooked sausage during the 45 days of storage at refrigerated temperature for lactic acid bacteria, and vegetative form of *C. prefringenes* and *C. prefringenes* spores and *B.coagulans* spores are displayed in Figure 1a, 1b, 1c, and 1d, respectively.

Lactic acid bacteria (LAB) are one of the main spoilage microorganisms in the refrigerated proteinaceous products such as cooked sausage (Korkeala & Björkroth, 1997). The initial LAB value of control sample was found to be 1.17 log CFU/g. According to Feng et al. (2013), upper microbiological limit of LAB for acceptable quality cooked sausage is 7 log CFU/g (Feng et al., 2013). As shown in Figure 1a, in control formulation 1, LAB reached 6.99 log CFU/g after 30 days of storage, and gradually increased to 8.5 log CFU/g at the end of the storage period. Microaerophilic environment and resistance of LAB to sodium nitrite, as well as low water activity of cooked sausage samples all together produced favorable conditions for growth of the psychotropic LAB strains (Díaz‐Vela, Totosaus, & Pérez‐Chabela, 2015). This result was in line with the findings reported by Rezaeigolestani et al. (2017) who noted values of 7.4 log CFU/g after 30 days of storage (Rezaeigolestani et al., 2017).

In vacuum‐packaged sausage, *Clostridia spp*. are the common organisms with potential spoilage and health hazard especially at prolonged storage times (Hernández‐Macedo et al., 2011). It is clear from the literature that even just one *Clostridia* spore may cause spoilage in vacuum meat products. In the current study, the initial count of inoculated *C. prefringenes* in sausage sample was 3.87 log CFU/g and their population decreased significantly (*p* < .05) approximately 2 log CFU/g in the treated samples compared with control formulation (Figure 1b). This significant decrease in *Clostridia* spp. population in treated sausage is consistent with the findings of Aminzare et al. (2018). The reduction in *C. prefringenes* population could be attributed to the strong antibacterial activity of thymol against pathogenic bacterial strains (Aminzare et al., 2018; Du et al., 2015).

There was a significant decrease in *C. prefringenes* population from 2.78 to 2.01 log CFU/g at the 120 ppm nitrite, 250 ppm thymol, and 450 ppm astaxanthin (Figure 1c). This relevant decrease in the present study for the highest level of thymol is in good agreement with the results showed by Juneja et al.(2007) who noted that the use of thymol resulted in a significant reduction in *C. perfringenes* spore growth during exponential cooling of contaminated cooked turkey up to 21 hr (Juneja & Friedman, 2007).

Finally, the results of the counts of inoculated *B. coagulans* spore‐forming bacteria in sausage samples during sampling days are summarized in Figure 1d. The obtained results showed a reduction in the spore count in the inoculated sausages. The lowest decrease rate was observed in control samples. This reduction in the count of *B. coagulans* spores was related to the effect of sausage ingredients especially those having impact on spore germination and outgrowth (such as the existence of sodium nitrite, salt, sodium ascorbate, thymol, and astaxanthin). However, the counts of the spores inoculated in samples were still above the recommended minimum daily therapeutic dose of spore probiotics (i.e., 10^6^ CFU/g) during the entire refrigerated storage (Jafari et al., 2017).

The absence of yeasts, molds, and Enterobacteriace has also been reported in other studies. The heat treatment, used in this study, proved to be effective and no postprocess contamination occurred due to the vacuum packaging. Because of the lack of *staphylococcus aureus*, hygienic procedures seem to have been effective and preventive. Previous studies have reported that the presence of sodium chloride and phosphate may be the cause of the inhibition in the growth of Enterobacteriace (Šojić et al., 2015; Viuda‐Martos, Ruiz‐Navajas, Fernández‐López, & Pérez‐Álvarez, 2010a, 2010b).

### 3.2 Chemical analysis

**Figure 2:**
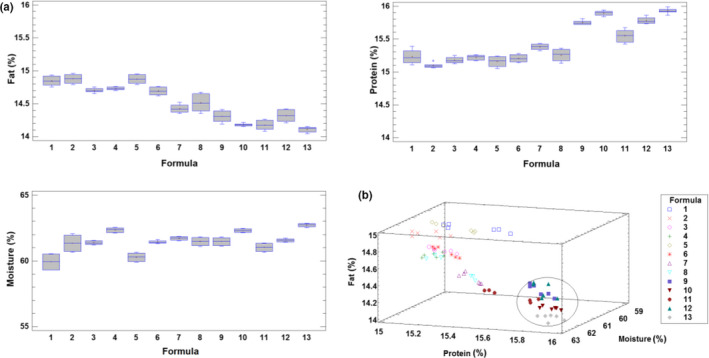
a) Box–whisker plots for the moisture, protein, and fat according to the different sausage formulations. b) 3D plot of moisture versus protein versus fat for the sausage samples according to the assayed formula

The results of the chemical analysis carried out in the samples of the different formulations for moisture, protein, and fat content at the end of the 45‐day storage period are summarized in Table 2. When an analysis of variance was carried out, for the three cases of moisture, protein, and fat, the results of the P‐value for the *F* test was less than 0.05, showing that there is a statistically significant difference between the averages of chemical variables among some of the different formulas, with a 95.0% confidence level. This is an expected result because, although the same raw materials were used for all formulations, these compositional parameters (moisture, protein, and fat) were slightly but clearly affected by the addition of natural antioxidants, antimicrobial compounds producing different microbial activity in the product, or in other words, the different quantities of astaxanthin, thymol, and nitrite, as well as the potential addition or not of *B. coagulans* leads to products with certain differences in chemical composition, and probably, as consequence, also in organoleptic properties. This fact has been illustrated in the box–whisker plots for these variables presented in Figure 2a, in which the chemical differences among formulations can be seen. The simultaneous visualization of these differences on the basis of each assayed formula was also examined in the xyz‐plot of the samples in Figure 2b. Specific chemical differences were detected for formulations 9–13 that perform a separate group with values of chemical variables distinct from the other formulations. The mean of moisture, fat, and protein contents ranged from 59.9% to 62.7%, 14.1% to 14.9%, and 15.0% to 15.9%, respectively.

**Table 2:** Results for the chemical analysis of the sausage samples according to the different formulations

Formula	Moisture (%)		Protein (%)		Fat (%)	
	Mean	S.D.	Mean	S.D.	Mean	S.D.
1	59.9	0.661	15.2	0.116	14.8	0.078
2	61.3	0.701	15.0	0.039	14.8	0.070
3	61.3	0.149	15.1	0.050	14.7	0.035
4	62.3	0.182	15.2	0.036	14.7	0.024
5	60.2	0.338	15.1	0.086	14.9	0.069
6	61.4	0.115	15.2	0.059	14.6	0.062
7	61.7	0.136	15.3	0.046	14.4	0.070
8	61.4	0.336	15.2	0.097	14.5	0.150
9	61.4	0.339	15.7	0.035	14.3	0.095
10	62.0	0.135	15.8	0.037	14.1	0.025
11	61.0	0.348	15.5	0.108	14.1	0.080
12	61.5	0.122	15.7	0.050	14.3	0.098
13	62.7	0.139	15.9	0.044	14.1	0.043

Abbreviation: S.D. standard deviation.

The effect of different concentrations of nitrite, thymol, and astaxanthin on the levels of TVN in the cooked sausage samples for the different formulations during the 45 days of storage at refrigerated temperature presented in Figure 1e. As shown in Figure 1e, TVN values increased, both in control and in treated samples, during the storage time; however, TVN values were significantly lower in the treated samples than in the control formulation (*p* < .05). The initial TVN for the control formulation 1 was 6.73 mg/100 g (comparable with previously reported results in Alirezalu et al. (2019) and Saleh et al. (2019) studies (Alirezalu et al., 2019; Saleh et al., 2019) and then reached to 30.63 mg/100 g after 45 days. In general, the lower and acceptable TVN values in the treated samples could be related to the antibacterial activities and the preservative effect of the additives evaluated. These compounds remarkably decreased the microbial growth and the activity of proteolytic enzymes in treated samples versus. control; the protein degradation into ammonia and amines also appreciably diminished (Du et al., 2019; Ghabraie et al., 2016; Weintraub et al., 2017).

On the other hand, lipid oxidation in cooked sausage reduces the quality during the storage period. One of the most common methods used in order to evaluate this oxidation is the determination of thiobarbituric acid reactive substances (TBARS). Malondialdehyde, the final product of lipid peroxidation of polyunsaturated fatty acids, is a reactive aldehyde that forms adduct with two thiobarbituric acid molecules to produce a pink color species absorbing at 532–535 nm. Thus, TBARS level is a good criterion for estimating the oxidative stress of the product. The evolution of TBARS measurements for the different formulations examined along the 45‐day period is presented in the Figure 1f. As seen, at the end of 45‐day study, TBARS levels varied between the most favorable cases (formulations 9–13) with values lower than 1 mg/kg up to the control formulation allowing TBARS content near 2 mg/kg. It is clear that the incorporation of thymol astaxanthin and nitrite significantly affect the level of lipid oxidation under the current experimental conditions. In general, the lower and acceptable level of TBARS in the treated sausages could be related to higher levels of nitrite (120 ppm) combined with high levels of thymol and astaxanthin. This fact could be explained due to radical scavenging activities of these substances reducing the lipid oxidation (Cheng & Wu, 2019; Luna et al., 2017; Weintraub et al., 2017). These results of TBARS in sausage samples during storage at refrigerated condition consistent with the findings reported in previous studies (Lee et al., 2019; Šojić et al., 2015).

Finally, the pH changes in the sausages with different formulations are displayed in Figure 1g. As seen, pH value of formulation 13 decreased to 6.59 at the end of the storage period which is in good agreement with previous study (Šojić et al., 2015). Also, in this present study the highest decrease rate was found (*p* < .05) in the control samples, in comparison with the other formulations. The growth of lactic acid bacteria (LAB) is responsible for changes in pH during storage of vacuum‐packaged sausages. Since the addition of thymol, nitrite, and astaxanthin may have affected the growth of LAB and reduced the production of lactic acid during storage (see Figure 1a), it seems reasonable to assume that this reduction can explain the slight pH decay during storage.

### 3.3 Chemometric analysis

In order to evaluate the joint influence of microbiological and chemical variables, the two chemometric display procedures described in section 2.4 were applied to a *X*_76x9_ data matrix in which the rows are the 76 sausage samples while the columns are the 9 variables corresponding to the chemical and microbiological variables at 45 days (the final day of storage period): moisture, fat, protein, pH‐45, *C. prefringes* spore count‐45, *C. prefringes* count‐45, lactic acid bacteria count‐45, TBAR‐45, and TVN‐45. Results from *Enterobacteriaceae* count as well as from yeasts and molds were not included since *Enterobacteriaceae* was not detected in none of the samples.

In order to avoid the influence of the different size of the variables in the chemometric analysis, all variables in the *X*_76x9_ data matrix were autoscaled, by subtracting each value from the mean of the variable and dividing it for the standard deviation of the variable. The result of *X*_AUT78x9_ matrix is a new matrix preserving the same chemical information than in the original one, but in this case all variables are of the same scale with 0 mean and 1 deviation standard (Deming et al., 1988). Therefore, latent relationships (between samples and between variables) and the internal structure residing in the *X*_AUT78x9_ data set were studied using two chemometric approaches: principal components analysis (PCA) and hierarchical cluster analysis (HCA).

**Figure 3:**
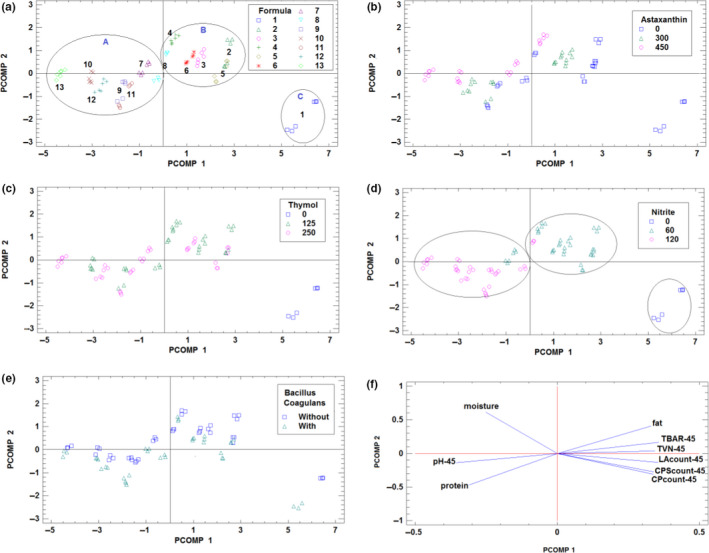
2D score plot obtained from PCA of the sausage samples according to the formulation (a), content of astaxanthin (b), content of thymol (c), content of nitrite (d), and addition or not of*B. coagulans*(e). 2D loading plot by PCA of the chemical and microbiological variables

**Figure 4:**
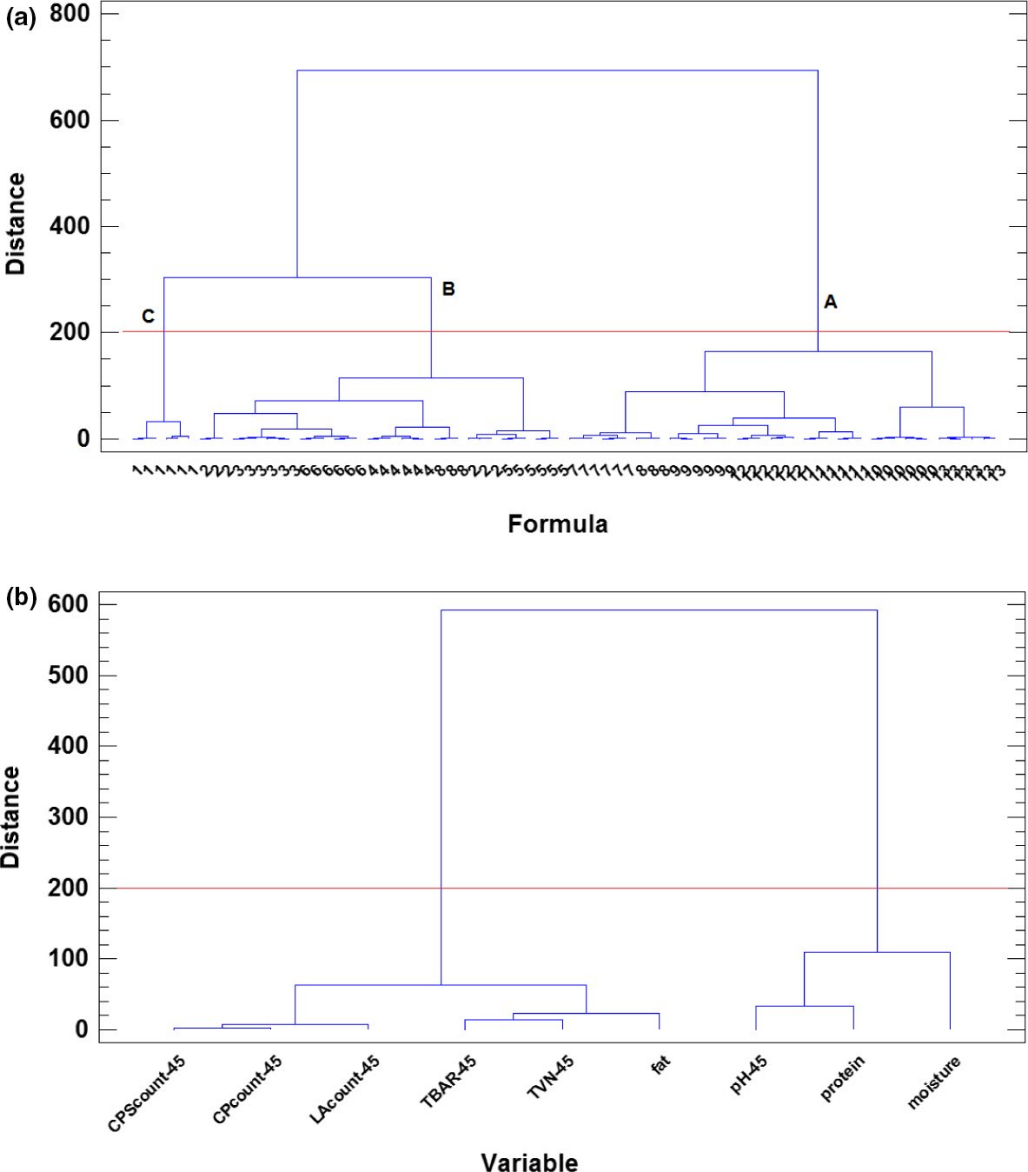
Dendrograms obtained by HCA (squared Euclidean distance, and Ward's agglomerative method) for samples (a) and variables (b) with different formulations

When PCA was applied to the autoscaled data matrix, the first two principal components were attained for the 91.24% of the total data variance. This means that the data can be studied in a 2‐dimensional space preserving more than the ninety percent of the total information contained in the data matrix. When the samples are evaluated in the 2D‐score plot of principal component 1, PCOMP 1 (representing 81.65% of the total data variance), versus. principal component 2, PCOMP 2 (accounting for the 9.59 of data variance), three groups of formulations were specified (see Figure 3a). The first one, named A, in the negative part of PCOMP 1, included samples from formulations 7–13. The second one, in the positive part of both principal components, is formed by the formulations 2–6, coded as B, and finally based on the formula 1 (as control sample), a single group C appeared which clearly is separated from the other formulations. In order to see the influence of the different preservatives added in the different formulations on chemical and microbiological variables jointly, in Figure 3b, 3c, and 3d, the samples in this score space were identified according to the content of astaxanthin, thymol, and nitrite, respectively. As it can be seen, the content of nitrite of the formulations is the more influential factor: Group C corresponds to formulations without nitrite added; group B includes formulations with nitrite concentration of 60 ppm, while group A is composed of the formulation with 120 ppm of nitrite. The only exception of this rule are samples of formula 7, that in spite contain 60 ppm, all of them appeared in group A, and this fact can be explained for synergistic antimicrobial activity with the other two compounds at high levels (thymol 250 ppm and astaxanthin 450 ppm). In addition, as it can be seen in Figure 3e, the addition of *B. coagulans* seems to clearly influence the joint set of chemical and microbiological variables, because for all formulations the samples with *B. coagulans* addition are placed at lower PCOMP 2‐scores. It can be concluded that *B. coagulans* as probiotic bacteria has antibacterial activity because its bacteriocin led to a reduction of spoilage microorganisms and consequently to a decrease in the protein degradation (Proteolysis). This result is consistent with several published studies which showed that synthesized extracellular polysaccharide by *B.coagulans* had significant antioxidant and free radical scavenging activities (Kodali & Sen, 2008). From the study of 2D loadings plot obtained from PCA (Figure 3f), it can be concluded that all of the microbiological variables are clearly related between them and to fat content of the samples while pH, protein, and moisture influence in the opposite direction. The increased fat content of the cooked sausage can be explained by the decrease in moisture which can increase other ingredients such as fat and also autolysis of lipoprotein to lipid and protein which lead to an increase in the amount of lipids (El‐Nashi et al., 2015; Yoon et al., 1997). The decrease in moisture content of sausage during refrigerated condition in control and samples containing lower amount of preservatives can be referred to as deterioration of proteins that are responsible for water holding capacity due to microbial growth in these treatments compared to treatments with higher concentration of preservatives. Samples with low concentration of nitrite, thymol, and astaxanthin that showed more decrease in pH during storage of sausages often related to the growth of lactic acid bacteria (LAB). The conclusions obtained by PCA were checked by applying other display chemometric technique with different mathematical base such as HCA. The results are presented in the dendrograms of Figure 4a and b, for the samples and variables, respectively. In the first dendrogram of the samples, the same clusters of formulations than the previous revealed by PCA were obtained (see Figure 4a). At a critical distance of 200, equal three A, B, and C clusters, each including the same formulations than in the previous step of chemometric analysis, were identified. Also, the appropriate confirmation for the relationship between variables explained by PCA has been verified through the dendrogram of variables in Figure 4b. Using HCA, the same association between variables that the previously revealed by PCA was obtained. The equivalence of the conclusions obtained by the two different chemometric techniques used (based on different mathematical approaches) corroborates their consistency.

### 3.4 Sensorial evaluation results

**Figure 5:**
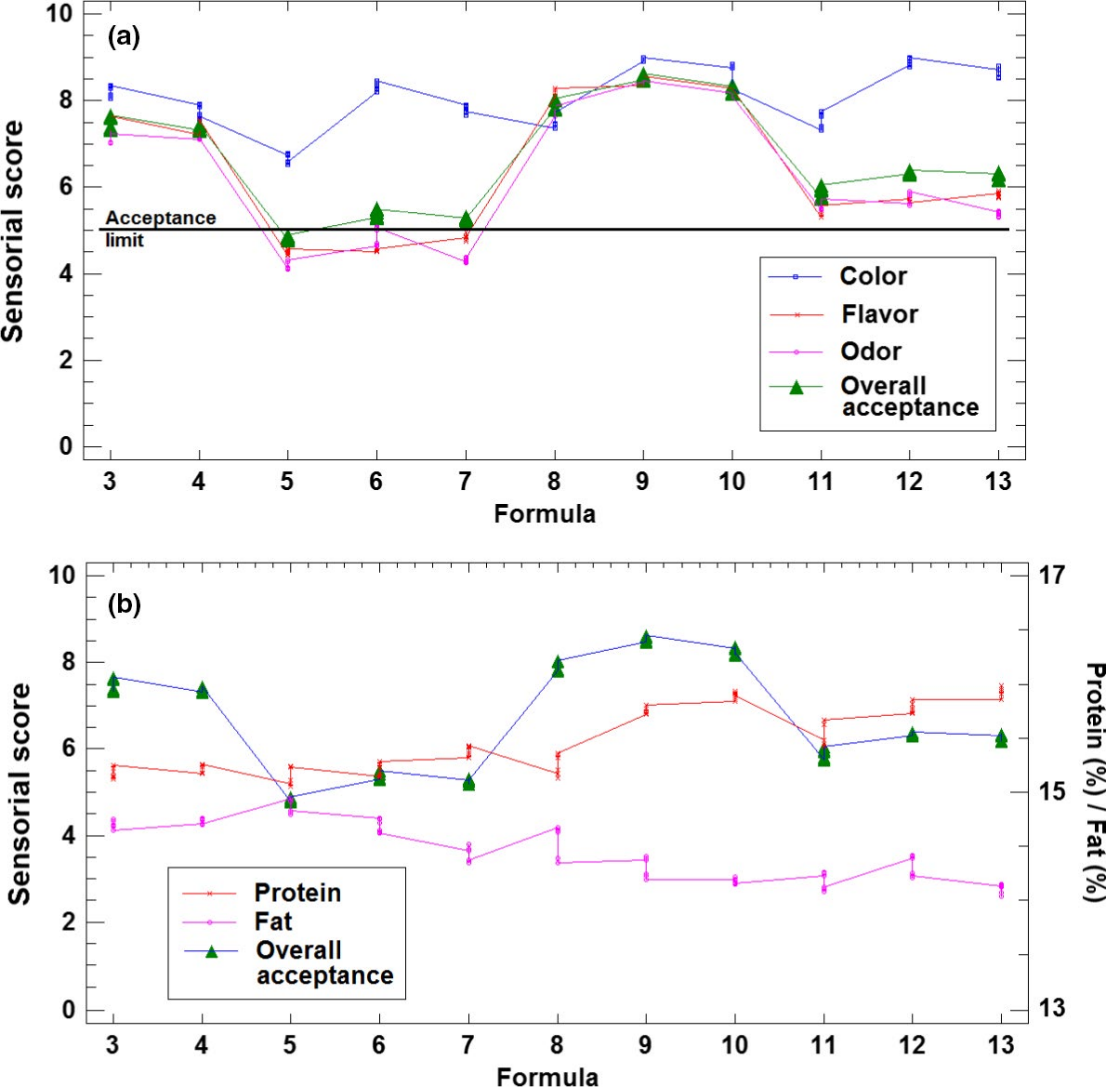
(a) Results of the sensorial analysis of the sausage samples of the different formulations. (b) Overall acceptance for the different sausage formulations in relation to protein and fat content

As indicated in section 2.3, the sausage samples prepared according to the different formulations subjected to a sensory evaluation carried out by an expert panel composed of 5 members. Results of this sensorial analysis on the basis of color, odor, flavor, and overall acceptance are presented in Figure 5a. The appropriate reproducibility of the results between replicates indicated the satisfactory work of panelists. As it can be seen in Figure 5, formula 5 exhibited sensorial scores lower than the acceptance limit for all the parameters evaluated. For formulas 6 and 7, in spite of the fact that their overall acceptance is slightly higher than 5, several sensorial parameters are below the acceptance limit. On the other hand, formulations 11, 12, and 13 also obtained low scores in the range 5 to 6. PCA, HCA, and TCATA are techniques used to analyze sensory evaluation (de Souza Paglarini et al., 2020; Vidal et al., 2019). According to PCA, treatments with thymol concentration (more than 250 mg/kg) have a low sensory score because thymol has a strong taste at high concentrations. On the other hand, formulations of 8, 9, and 10 which had obtained highest scores by panelist, all had low level of thymol (125 ppm), high level of Nitrite (120 ppm), and different quantities of astaxanthin between 0 and 450 ppm. In addition, as seen in Figure 5b, the overall acceptance score is also studied in relation to the protein and fat content. As it can be seen, the most valuable formulations in terms of sensorial score (formulas 8, 9, and 10) are those corresponding to slightly high‐protein and slightly low‐fat levels in comparison with the remaining formulas. According to the findings reported in previous studies, the use of probiotics in food can reduce the deterioration of sensory properties of food products (Karimi et al., 2012).

### 3.5 Sausage formulation selection

The selection of the better formulation was carried out by taking into account both chemical and microbiological analyses, as well as the results from sensory data. On the basis of microbiological and chemical determinations, among the formulations of cluster A, the formulation 13 (with maximum levels assayed for astaxanthin, thymol, and nitrate) should be selected because it allows for better results as indicated in the text and can easily be checked in Figure 3a. However, when the results of sensory data were also considered, formulations 8, 9, and 10 were preferred (Figure 5a). By combining the two criteria, formulation 10 must be selected as optimum because (i) relation to the chemical and microbiological characteristics is highly similar, in practice, to the best one to be selected following this criterium, formula 13. In fact, the near position of the samples of both formulations 10 and 13 in the PCA score plot in Figure 3a demonstrate this statement; and (ii) on the basis of sensory data, formula 10 also belongs to the set of formulations (8, 9, and 10) clearly preferred by tasters. Thus, formula 10 (astaxanthin 450 ppm, thymol 125 ppm, and nitrite 120 ppm) was selected as optimum for future developments.

## 4 CONCLUSION

In summary, among the different treatments in this study, and among sausages formulated containing different concentrations of nitrite, thymol, and astaxanthin, a promising approach was suggested for limiting microbial growth and decreasing TBARS values in the sausages. Furthermore, a synergistic antibacterial effect observed between thymol and nitrite, with the highest concentrations of thymol, astaxanthin, and nitrite showed the best antibacterial activity. However, high concentration of thymol leads to adverse organoleptic properties. Based on our findings, it can be argued that integration of thymol and astaxanthin into the sausage formulation successfully improved the quality of beef cooked sausage during storage in refrigerator condition. The evidence from this study and similar studies suggests that thymol and astaxanthin can play a favorable role in enhancing cooked sausage quality.

## 5 CONFLICT OF INTEREST STATEMENT

The researchers declare that there are no conflicts of interest.
